# SYVN1, an ERAD E3 Ubiquitin Ligase, Is Involved in GABA_A_α1 Degradation Associated with Methamphetamine-Induced Conditioned Place Preference

**DOI:** 10.3389/fnmol.2017.00313

**Published:** 2017-10-05

**Authors:** Dong-Liang Jiao, Yan Chen, Yao Liu, Yun-Yue Ju, Jian-Dong Long, Jiang Du, Chang-Xi Yu, Yu-Jun Wang, Min Zhao, Jing-Gen Liu

**Affiliations:** ^1^Collaborative Innovation Center for Brain Science, Shanghai Mental Health Center, Shanghai Jiao Tong University School of Medicine, Shanghai, China; ^2^Department of Psychiatry, Bengbu Medical College, Bengbu, China; ^3^Department of Pharmacology, College of Pharmacy, Fujian Medical University, Fuzhou, China; ^4^College of Pharmacy, Fujian University of Traditional Chinese Medicine, Fuzhou, China; ^5^Key Laboratory of Receptor Research, Shanghai Institute of Materia Medica and Collaborative Innovation Center for Brain Science, Chinese Academy of Sciences, Shanghai, China; ^6^Shanghai Key Laboratory of Psychotic Disorders, Shanghai Mental Health Center, Shanghai Jiao Tong University School of Medicine, Shanghai, China

**Keywords:** methamphetamine, GABA_A_α1, dorsal striatum, ERAD, SYVN1

## Abstract

Abuse of methamphetamine (METH), a powerful addictive amphetamine-type stimulants (ATS), is becoming a global public health problem. The gamma-aminobutyric acid (GABA)ergic system plays a critical role in METH use disorders. By using rat METH conditioned place preference (CPP) model, we previously demonstrated that METH-associated rewarding memory formation was associated with the reduction of GABA_A_α1 expression in the dorsal straitum (Dstr), however, the underlying mechanism was unclear. In the present study, we found that METH-induced CPP formation was accompanied by a significant increase in the expression of Synovial apoptosis inhibitor 1 (SYVN1), an endoplasmic reticulum (ER)-associated degradation (ERAD) E3 ubiquitin ligase, in the Dstr. The siRNA knockdown of SYVN1 significantly increased GABA_A_α1 protein levels in both primary cultured neurons and rodent Dstr. Inhibition of proteasomal activity by MG132 and Lactacystin significantly increased GABA_A_α1 protein levels. We further found that SYVN1 knockdown increased GABA_A_α1 in the intra-ER, but not in the extra-ER. Accordingly, endoplasmic reticulum stress (ERS)-associated Glucose-regulated protein 78 (GRP78) and C/EBP homologous protein (CHOP) increased. Thus, this study revealed that SYVN1, as the ERAD E3 ubiquitin ligase, was associated with Dstr GABA_A_α1 degradation induced by METH conditioned pairing.

## Introduction

Amphetamine-Type Stimulants (ATS), the second-most prevalent illicit drugs after cannabis/marijuana in the world (United Nations Office on Drugs and Crime (UNODC), 2011), are highly addictive and can lead to psychiatric illnesses or cognitive defect (Batki and Harris, 2004; Curran et al., 2004). ATS include amphetamine, methamphetamine (METH), 3,4-methylenedioxyamphetamine (MDA), and 3,4-methylenedioxymethamphetamine (MDMA), among which METH is the most potent amphetamine derivative and widely used substance. The mechanism underlying METH dependence is poorly understood.

Emerging increasing evidence shows that GABAergic dysfunction plays a critical role in the development of ATS use disorders (Addolorato et al., 2012; Kumar et al., 2013; Jiao et al., 2015). The GABA_A_ receptors are the major inhibitory receptors in the central nervous system. Use of ATS will damage the normal functions of GABA_A_ receptors (Hondebrink et al., 2013), and GABA_A_ receptor modulators are reported to be effective in treating ATS use disorders (Mintzer and Griffiths, 2003; Rush et al., 2004; Johnson et al., 2007; Spence et al., 2016; Berro et al., 2017). The GABA_A_ receptors are ligand-gated and made up of five protein subunits that belong to different subunit classes, including α1–6, β1–3, γ1–3, δ, ε, θ1–3, π, ρ1–3. Our previous study demonstrated that the level of GABA_A_α1 subunits was significantly decreased in the dorsal striatum (Dstr). We also found that the GABA_A_α1 expression level in the Dstr decreased only in METH pairing group, but remained unchanged in the unpaired group, indicating that the changes of GABA_A_α1 receptors were related to METH-pairing, rather than the pure drug effects. Intra-Dstr injection of the specific α1-containing GABA_A_ receptor agonist before pairing abolished METH-induced conditioned place preference (CPP) formation. Therefore, our findings demonstrated that the decrease of GABA_A_α1 proteins in the Dstr was correlated to METH rewarding memory formation (Jiao et al., 2016). So far, little was known about the underlying mechanism of this decreased GABA_A_α1 expression associated with METH pairing.

Endoplasmic reticulum (ER)-associated degradation (ERAD) is the process by which the ER directs the degradation of misfolded or inappropriate proteins. It has been reported that METH or cocaine abuse is able to increase the misfolded or inappropriate proteins in ER and induce endoplasmic reticulum stress (ERS; Jayanthi et al., 2004, 2009; Beauvais et al., 2011; Pavlovsky et al., 2013). Moreover, it has been demonstrated that ERAD is involved in GABA_A_α1 degradation (Kang and Macdonald, 2004; Gallagher et al., 2005). Thus, we hypothesized that Dstr GABA_A_α1 protein was degraded via ERAD in METH dependent rat. In this study, by utilizing METH-induced CPP model, we tested this hypothesis by investigating the role of ERAD in the regulation of GABA_A_α1 expression, and found that Synovial apoptosis inhibitor 1 (SYVN1), the ERAD E3 ubiquitin ligase, modulated the downregulation of GABA_A_α1 protein associated with METH CPP.

## Materials and Methods

### Animals

Sprague Dawley male rats (weighting 220–300 g) were purchased from the Experimental Animal Center, Chinese Academy of Sciences (Shanghai, China). Rats were housed 2–3 per cage on a 12/12-h light/dark cycle with free access to food and water. All the animals used in this study were maintained in accordance with the National Institutes of Health Guide for the Care and Use of Laboratory Animals (NIH Publications No. 80-23, revised 1996) and experiments were approved by the Institutional Animal Care and Use Committee of Chinese Academy of Sciences (Shanghai, China).

### Drugs and Antibodies

METH was provided by China Academy of Military Medical Science. The primary antibodies include anti-GABA_A_α1 (1:1000, Millipore), anti-GABA_A_α2 (1:1000, Millipore), anti-GABA_A_α3 (1:1000, Santa Cruz), anti-GABA_A_α5 (1:1000, Abcam), anti-GABA_A_β2 (1:1000, Millipore), anti-SYVN1 (1:1000, Abcam), anti-Gp78 (1:1000, Abcam), Anti-CHOP (1:1000, Abcam), Glucose-regulated protein 78 (GRP78; 1:1000, Abcam), anti-Ubiquitin (1:1000, Cell Signaling) and anti-Glyceraldehyde-3-Phosphate Dehydrogenase (GAPDH; 1:20,000, Bio Legend).

### Conditioned Place Preference

The CPP apparatus (Jiliang Software and Instruments) consists of two square compartments of the same size [40 cm (length) × 40 cm (width) × 60 cm (height)], separated by removable doors (10 × 10 cm), allowing rats free access to each compartment. One compartment has black and white horizontal stripes walls with an iron wire floor and the other has black and white vertical stripes walls with a steel bar floor.

CPP procedure has been described in our previous studies (Jiao et al., 2016). The place conditioning procedure consists of the following phases: Habituation, Preconditioning, Conditioning and Testing. During Habituation phase, rats were allowed access to the entire apparatus for 30 min. In the Preconditioning phase, rats were allowed access to both compartments of the apparatus for 15 min. The duration spent in each compartment was recorded. The animals showing strong unconditioned aversion (one compartment >720 s) for one of the compartments were excluded. Following the Preconditioning, rats went through 8 days of conditioning. On the first day, the rats were intraperitoneally injected with either METH (1 mg/kg,) or saline (1 mL/kg.) and then confined to the non-preferred compartment for 45 min. On alternate days, rats were received saline and placed immediately in the preferred compartment. The procedure was repeated four times in the conditioning phase. During Testing phase, 24 h after the conditioning trial, the doors were opened and the rats were allowed to explore the entire apparatus for 15 min. The time spent in each compartment was recorded. The CPP score was the time spent in the drug-paired compartment during the testing phase minus the time spent in that compartment during the preconditioning phase. To minimize the basal stress, all rats were gentled by handling twice a day till the start of behavioral experiment. METH treated rats and saline-treated rats were individually housed in different cages with same standard conditions.

### Subcellular Fractionation

Rats were sacrificed and the brains were removed immediately after CPP conditioning. The brain was dissected into coronal slices (1 mm thick) using a rat brain slicer (Braintree Scientific), and the dorsal striatum was punched by a bluntend, 17-gauge syringe needle. The homogenate was centrifuged at 1000× *g* for 10 min at 4°C, and the supernatant was collected for analysis.

### Immunoblotting

Equal amounts of protein were loaded on 10% sodium dodecyl sulfate polyacrylamide gels for electrophoresis. Separated proteins were then transferred on nitrocellulose membrane. GABA_A_α1, GABA_A_α2, GABA_A_α3, GABA_A_α5, GABA_A_β2, Gp78, SYVN1, C/EBP homologous protein (CHOP) or GRP78 was detected with primary antibody at a 1:1000 dilution in TBS containing 5% non-fat dried milk and 0.05% Tween-20. After incubation with secondary antibody (HRP-conjugated goat anti-rabbit IgG or goat anti-mouse IgG) at a 1:2000 dilution, immunoreactive bands were detected with chemiluminescent substrate (RPN2232, GE Healthcare). The immunoreactive signals were quantified by quantity analysis software (Bio-Rad).

### Co-Immunoprecipitation (Co-IP)

Brain homogenate was incubated overnight with antibodies (anti-GABA_A_α1 or anti-SYVN1; dilution 1:100) and with Protein A or Protein G agarose beads (Sigma) at 4°C. Beads were washed three times by centrifugation and bound proteins were analyzed by Western blotting.

### Quantitative Reverse Transcriptase PCR (qRT-PCR)

Total RNA was isolated from rat Dstr using a commercially available kit (RNeasy Plus Mini Kit, Qiagen). QRT-PCR was performed on a ABI7500 Real-Time PCR system (ABI) using SYBR^®^ Premix Ex Taq™ kit (TaKaRa Bio Group, Japan). A typical reaction of a total volume of 20 μl consisted of 2 μl Template DNA, 10 μl 2× SYBR Green Reaction Mix, 0.4 μl PCR Forward Primer (10 μM), 0.4 μl PCR Reverse Primer (10 μM), 0.4 μl ROX Reference Dye II and 6.8 μl DEPC treated water. PCR amplification was done with an initial incubation at 95°C for 30 s, and then followed by 40 cycles of 95°C for 5 s, 60°C for 34 s, and final melting curve from 95°C for 5 s, 60°C 60 s. Primer specificity was confirmed by melting curve analysis. The mRNA for GABA_A_α1 and SYVN1 was normalized to a control gene (GAPDH). Primers utilized were as follows: GABA_A_α1 (GABA_A_α1, Fwd—CCTGGACCCTCATTCTGAGCA, Rev—ATCCTCGTGAAGACAGTGGTGTTG; GAPDH, Fwd—AACTTTGGCATTGTGGAAGG, REV—ACACATTGGGGGTAGGAACA; Genewiz, China).

### Intracerebral Microinjection

Rats were anesthetized using sodium pentobarbital (50 mg/kg, i.p.) under aseptic conditions, and then a stereotaxic instrument (Narishige) with the incisor bar set at 3.3 mm was used for Cannula implantation and microinjection. For dorsal striatum infusion, guide cannula (26 gauge) was bilaterally implanted in the dorsal striatum (anteroposterior: +0.6 mm; mediolateral: ±2.7; dorsoventral: −2.0 mm). After recovering from surgery and anesthesia for 1 week, animals were placed in a stereotaxic apparatus again for drug infusion. Bilateral microinfusions were made through 31-gauge injection needles (2 mm below the tip of the guide cannulae), which was connected to a 10 μl microsyringe mounted in the microinfusion pump (Harvard Apparatus). During the injection, the animals were gently restrained by hand.

Mg132 and Lactacystin were dissolved in dimethylsulfoxide (DMSO) to stock concentration (50 μg/μl) and then diluted in PBS to a work concentration (0.25 μg/μl, DMSO concentration ≤20%). During the conditioning session, 10 min before METH administration (1 mg/kg, i.p.), Mg132 or Lactacystin (0.5 μg/side) was bilaterally microinjected into the dorsal striatum. Each infusion volume was 2 μl per side infused at a rate of 0.4 μl/min. After injection, rats were given an additional 2 min to allow drug diffusion.

### Histology

Animals were deeply anesthetized with sodium pentobarbital and received a transcardial perfusion with 0.9% saline followed by 4% paraformaldehyde in PBS. The brains were removed and post fixed in 4% paraformaldehyde for 24 h and then kept in a 30% sucrose/PBS solution for 3–5 days to dehydration. The brains were cut coronally in 30 μm thickness on a cryostat (Leica). The tissue was stained with cresyl violet, and examined by light microscopy to detect the injection sites. Only animals with right injection sites were included for data analysis.

### Lentivirus and Adeno-Associated Virus (AAV)

The SYVN1 lentivirus and adeno-associated virus (AAV) were purchased from Obio Technology (Shanghai) Co., Ltd. The lentiviral stocks (1.5–2.0 × 10^8^ integration units (IU)/ml) were used to infect cultured neurons. The fluorescence was examined and the lysates were collected at 96 h after infection.

The AAV stocks (3.0–3.5 × 10^12^ IU/mL) were used to infect rodent Dstr. Rats were anesthetized and the guide cannula (26 gauge) was bilaterally implanted in the dorsal striatum (anteroposterior: +0.6 mm; mediolateral: ±2.7; dorsoventral: −2.0 mm). Bilateral microinfusions were made through 31-gauge injection needles (2 mm below the tip of the guide cannulae), connecting to a 10 μl microsyringe mounted in the microinfusion pump (Harvard Apparatus). AAV (1.5 μl/site) was microinjected into the dorsal striatum over 15 min. The microinjection needle was retained for another 5 min for drug diffusion. Two to three weeks after AAV-injection, animals were deeply anesthetized and received a transcardial perfusion. The brains were collected, dehydrated and cut for fluorescence examining.

### Endoplasmic Reticulum Isolation

The ER was isolated from rat dorsal striatum using a commercially available kit (Endoplasmic Reticulum Isolation Kit, Sigma) according to the supplier’s instructions. Isolated ER was stored at −20°C for analysis.

### Data Analysis

The data were analyzed with one-way or two-way analysis of variance (ANOVA), and then followed by Newman-Keuls or Bonferroni *post hoc* tests. Differences with *p* < 0.05 were considered statistically significant. The results are presented as mean ± SEM.

## Results

### Decrease in GABA_A_α1 Protein Expression in the Dstr of METH-CPP Rat

**Figure 1:**
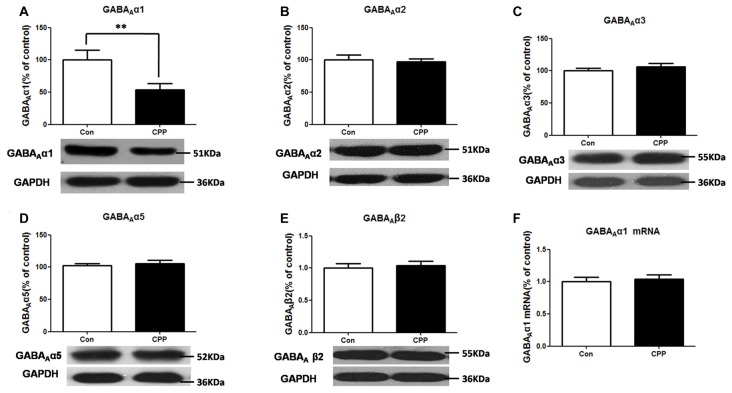
Decrease in GABA_A_α1 protein expression in the dorsal straitum (Dstr) of methamphetamine-conditioned place preference (METH-CPP) rat.** (A)** Dstr GABA_A_α1 protein expression level decreased in METH-CPP rat. **(B–E)** There were no changes of the expression levels of GABA_A_α2 **(B)**, GABA_A_α3 **(C)**, GABA_A_α5 **(D)** and GABA_A_β2 **(E)** after METH pairing. **(F)** mRNA was determined by qRT-PCR, and the values were normalized to the geometric mean of a control gene (Glyceraldehyde-3-Phosphate Dehydrogenase (GAPDH). Error bars represent mean ± SEM, *n* = 5–8 in each group. **p < 0.01 compared with control group, two tailed Student’s *t*-test.

We previously found that conditioned METH rewarding reduced GABA_A_α1 protein expression in the Dstr. This result was confirmed in the present study (*p* < 0.01 vs. control group; Figure 1A). And we did not find any significant changes in the other subunits protein levels of GABA_A_α2, GABA_A_α3, GABA_A_α5 and GABA_A_β2 in Meth-CPP rat (*p* > 0.05; Figures 1B–E). We further detected the GABA_A_α1 mRNA expression in the Dstr, and we found that there were no expression changes of the GABA_A_α1 mRNA (*p* > 0.05; Figure 1F). These results indicated that GABA_A_α1 levels were lower in the Dstr after METH pairing, and it occurred at the post-translational level.

### Increased SYVN1 Protein Expression in the Dstr of METH-CPP Rat

**Figure 2:**
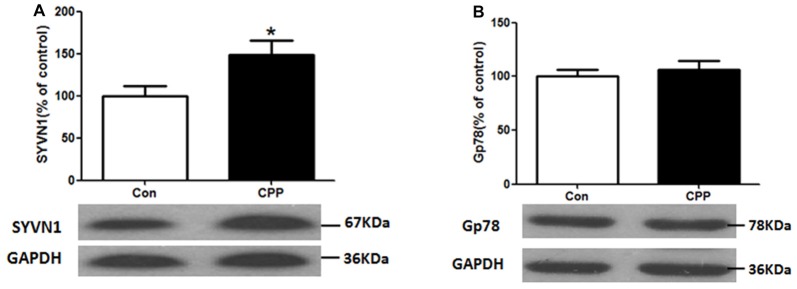
Increase in Synovial apoptosis inhibitor 1 (SYVN1) protein expression in the Dstr of METH-CPP rat. **(A)** SYVN1 protein expression level increased in the Dstr. **(B)** Glycoprotein 78 (Gp78) protein expression level remained unchanged. Error bars represent mean ± SEM, *n* = 8 in each group. *p < 0.05 compared with control group, two tailed Student’s *t*-test.

ERAD has been reported to be involved in the degradation of un- or misfolded GABA receptors (Kang and Macdonald, 2004; Gallagher et al., 2005). The mammalian ERAD systems are organized primarily by two E3 ubiquitin ligases: SYVN1 and Glycoprotein 78 (Gp78; Christianson et al., 2011). We thus examined the protein expression levels of SYVN1 and Gp78, and we found that the SYVN1 level, but not Gp78 level, was significantly increased (*p* < 0.05 vs. control group; Figure 2). These results suggested that the SYVN 1 might be a key component that associated with degradation of GABA_A_α1.

### SYVN1 Was Associated with GABA_A_α1 Degradation

**Figure 3:**
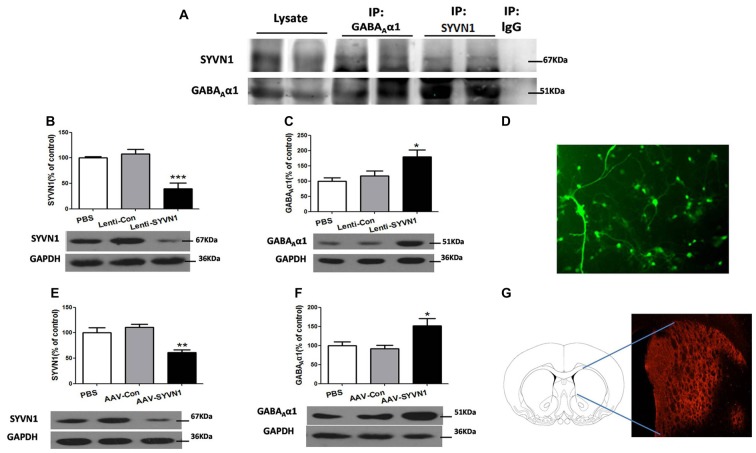
SYVN1 was associated with GABA_A_α1 degradation.** (A)** GABA_A_α1 co-precipitated with SYVN1. Lysates from Dstr were subjected to co-immunoprecipitation (Co-IP) using an anti-GABA_A_α1 antibody followed by western blotting with SYVN1 or GABA_A_α1 antibody. In reverse IP, lysates were immunoprecipiated with an anti-SYVN1 antibody followed by western blotting with GABA_A_α1 or SYVN1 antibody. **(B)** The SYVN1 expression level was significantly reduced in primary striatum neurons transfected with the Lenti-SYVN1. **(C)** Knockdown of SYVN1 by Lenti-SYVN1 increased GABA_A_α1 protein level in primary striatum neurons. **(D)** Representative image of *in vitro* lentivirus-infected primary neurons visualized by fluorescence microscope. **(E)** The SYVN1 expression level was significantly reduced in rat Dstr transfected with the adeno-associated virus (AAV)-SYVN1. **(F)** Knockdown of SYVN1 by AAV-SYVN1 increased GABA_A_α1 protein levels in rat Dstr. **(G)** Representative image of *in vivo* AAV-infected Dstr visualized by fluorescence microscope. Error bars represent mean ± SEM, *n* = 4–6 in each group. ***p < 0.001, **p < 0.01, *p < 0.05 compared with the corresponding control groups, one-way analysis of variance (ANOVA) with Newman-Keuls *post hoc* test.

We sought to investigate whether SYVN1 could interact with GABA_A_α1 and be responsible for the GABA_A_α1 degradation. As shown in Figure 3A, SYVN1 was detected in GABA_A_α1 immunoprecipitates, but not in the control IgG. The interaction was further verified using a reciprocal approach, where GABA_A_α1 co-purified with SYVN1, but not with a control IgG. The data indicated that GABA_A_α1 interacted with SYVN1 in the Dstr of METH pairing rat.

We then used SYVN1-shRNA to examine the causal relationship between SYVN1 and GABA_A_α1 degradation. Infection of primary Dstr neurons with Lenti-SYVN1 (Figure 3D) significantly decreased SYVN1 expression level (PBS, 100 ± 2.713%; Lenti-Con, 107.4 ± 9.653%; Lenti-SYVN1, 39.25 ± 11.20%; *F*_(2,11)_ = 18.58, *p* = 0.0006; Figure 3B), indicating the effectiveness of the lentivirus system. As shown in Figure 3C, SYVN1 knockdown resulted in a significant increase in GABA_A_α1 expression (PBS, 100 ± 11.02%; Lenti-Con, 116.8 ± 17.10%; Lenti-SYVN1, 180.0 ± 21.74%; *F*_(2,11)_ = 6.025, *p* = 0.0218).

In order to improve the *in vivo* viral infection efficiency, we used AAV expressing shRNA (AAV-SYVN1). We found that infection of striatum neurons with AAV-SYVN1 (Figure 3G) significantly decreased SYVN1 expression level (PBS, 100 ± 10.27%; AAV-Con, 110.4 ± 6.599%; AAV-SYVN1, 61.23 ± 5.510%; *F*_(2,17)_ = 11.22, *p* = 0.0010; Figure 3E). As shown in Figure 3F, SYVN1 knockdown significantly increased GABA_A_α1 protein levels (PBS, 100 ± 9.781%; AAV-Con, 91.80 ± 8.823%; AAV-SYVN1, 152.0 ± 19.13%; *F*_(2,17)_ = 5.918, *p* = 0.0127), which was consistent with our *in vitro* data. These results indicated that SYVN1 was involved in regulating GABA_A_α1 degradation.

### The Ubiquitin-Proteasome System (UPS) Was Involved in the Degradation of GABA_A_α1

**Figure 4:**
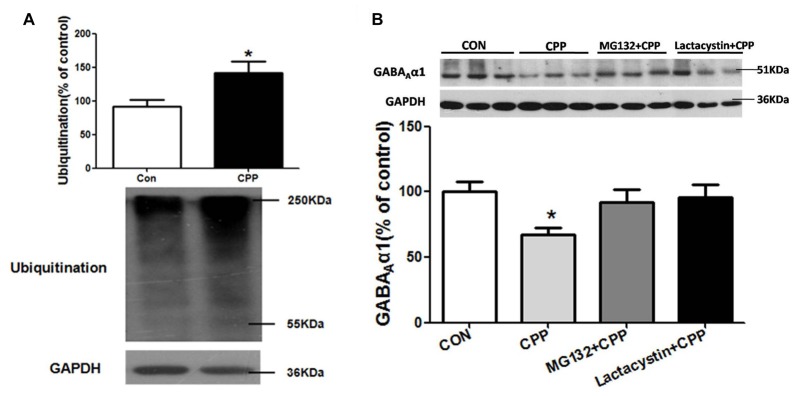
Ubiquitin-proteasome system (UPS) mediated GABA_A_α1 degradation.** (A)** Ubiquitin protein expression level increased in the Dstr of METH-CPP rat. Error bars represent mean ± SEM, *n* = 7 in each group. *p < 0.05 compared with control group, two tailed Student’s *t*-test. **(B)** Intra-Dstr injection of proteasomal inhibitor MG132 and Lactacystin reversed the reduced of GABA_A_α1 induced by METH-CPP. Error bars represent mean ± SEM, *n* = 7 in each group. *p < 0.05 compared with the corresponding control groups, one-way ANOVA with Newman-Keuls *post hoc* test.

SYVN1, as an E3 ubiquitin ligase, significantly increased in the Dstr, suggesting that ubiquitin-proteasome system (UPS) might be involved in METH-induced CPP formation. We thus tested the level of ubiquitin protein in Dstr of METH-CPP rat, and we found that the expression level of ubiquitin protein was remarkably enhanced after conditioned METH pairing (*p* < 0.05 *vs* control group; Figure 4A). We further found that intra-Dstr injection of proteasomal inhibitor MG132 and Lactacystin could reverse the change of GABA_A_α1 expression induced by METH pairing (Con, 100 ± 7.408%; CPP, 68.14 ± 6.145%; MG132+CPP, 92.10 ± 9.942%; Lactacystin+CPP, 95.73 ± 9.668%; *F*_(3,27)_ = 3.034, *p* = 0.0497; Figure 4B). These data demonstrated that UPS, as part of ERAD, was involved in the degradation of GABA_A_α1.

### *In Vivo* Knockdown of Dstr SYVN1 Increased the Expression Level of Glucose-Regulated Protein 78 (GRP78) and C/EBP Homologous Protein (CHOP) in METH-CPP Rat

**Figure 5:**
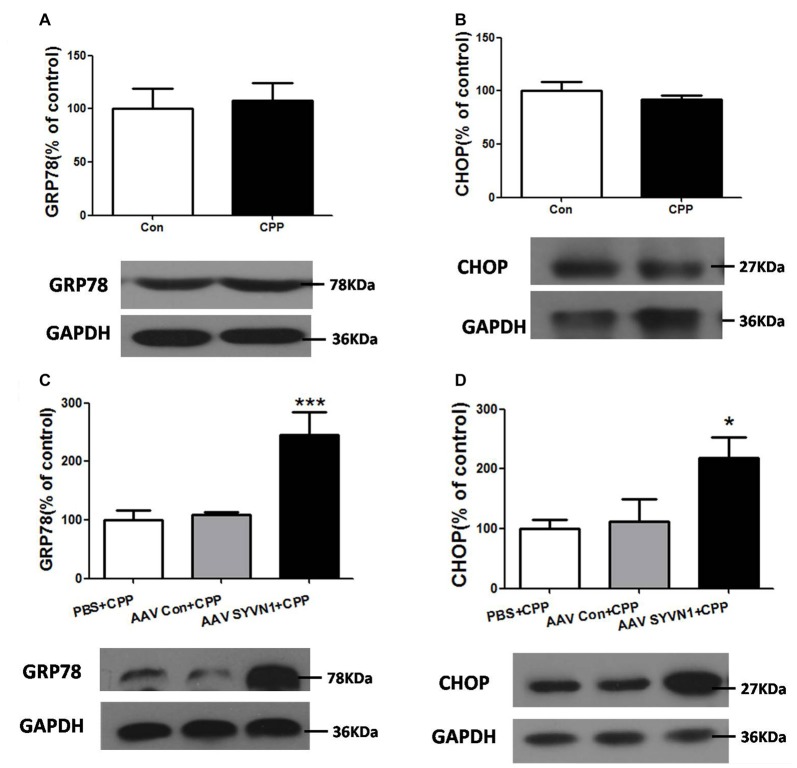
Knockdown of Dstr SYVN1 increased the level of Glucose-regulated protein 78 (GRP78) and C/EBP homologous protein (CHOP) in METH-CPP rat.** (A,B)** Conditioned METH pairing did not affect Dstr GRP78 and CHOP expression level. Error bars represent mean ± SEM, *n* = 6 in each group. *p* > 0.05 compared with the control group, two tailed Student’s *t*-test. **(C,D)** Knockdown of SYVN1 increased Dstr GRP78 and CHOP levels in METH-CPP rat. Error bars represent mean ± SEM, *n* = 6 in each group. ***p < 0.001, *p < 0.05 compared with the corresponding control groups, one-way ANOVA with Newman-Keuls *post hoc* test.

Increased misfolded and unassembled membrane proteins may induce ERS, which is always associated with increased expression of the ER resident chaperone GRP78 and CHOP. We thus examined the expression level of GRP78 and CHOP in the Dstr of METH pairing rat, and we found there were no changes of either GRP78 or CHOP compared with the control group (*p* > 0.05, Figures 5A,B). However, *in vivo* knockdown of Dstr SYVN1 significantly increased the level of GRP78 (PBS+CPP, 100 ± 16.84%; AAV Con + CPP, 108.6 ± 4.813%; AAV SYVN1+CPP, 246.1 ± 37.74%; *F*_(2,17)_ = 11.65, *p* = 0.0009; Figure 5C) and CHOP (PBS+CPP, 100 ± 14.87%; AAV control + CPP, 111.7 ± 37.29%; AAV SYVN1 + CPP, 218.2 ± 35.47%; *F*_(2,17)_ = 4.438, *p* = 0.0306; Figure 5D) in Dstr of METH-CPP rat. These results suggested that knockdown of Dstr SYVN1 might make misfolded or inappropriate GABA_A_α1 proteins subunit failed to degradate through ERAD and accumulated in ER, which triggered ERS.

### Knockdown of SYVN1 Increased the GABA_A_α1 Protein Expression in the Intra-ER Rather than Extra-ER

**Figure 6:**
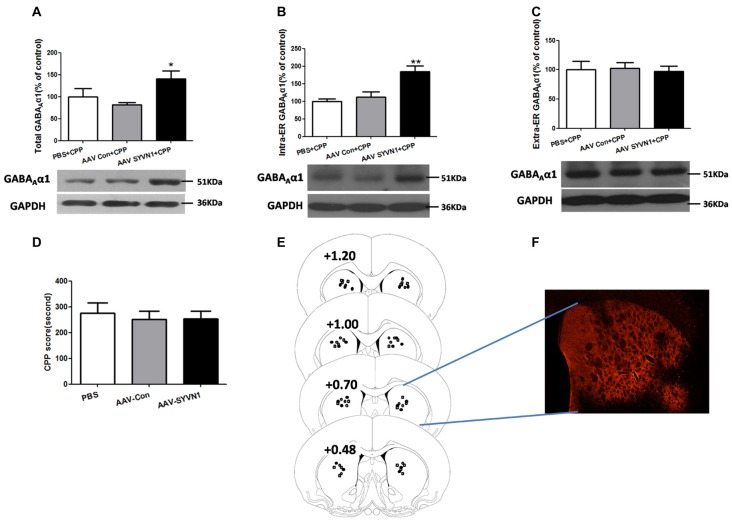
Knockdown of SYVN1 increased the GABA_A_α1 protein expression in the intra-ER rather than extra-ER in the METH-CPP rat.** (A)** Knockdown of Dstr SYVN1 increased total GABA_A_α1 protein. **(B)** Knockdown of Dstr SYVN1 increased GABA_A_α1 protein in the intra-ER. **(C)** Knockdown of Dstr SYVN1 protein did not affect GABA_A_α1 protein in the extra-ER. **(D)** Knockdown of Dstr SYVN1 protein did not affect METH-CPP formation. **(E)** Schematic representation of injection sites in the Dstr. **(F)** Representative image of *in vivo* AAV-infected Dstr visualized by fluorescence microscope. Error bars represent mean ± SEM. *n* = 6–11 in each group. **p < 0.01, *p < 0.05, compared with the corresponding control groups, one-way ANOVA with Newman-Keuls *post hoc* test. (○, PBS; □, AAV-Con; •, AAV-SYVN1).

We further isolated ER and examined the expression level of GABA_A_α1 in the intra-ER and extra-ER. We used AAV-SYVN1 to infect striatum neurons (Figures 6E,F) and found that knockdown of Dstr SYVN1 increased the expression level of total GABA_A_α1(PBS+CPP, 100 ± 18.68%; AAV Con+CPP, 81.33 ± 5.623%; AAV SYVN1+CPP, 140.9 ± 17.67%; *F*_(2,17)_ = 4.018, *p* = 0.0400; Figure 6A) and GABA_A_α1 in the intra-ER (PBS+CPP, 100 ± 7.488%; AAV Con+CPP, 111.8 ± 15.08%; AAV SYVN1+CPP, 184.3 ± 16.44%; *F*_(2,17)_ = 11.29, *p* = 0.0010; Figure 6B), but not in the extra-ER (PBS+CPP, 100 ± 14.39%; AAV Con+CPP, 102.2 ± 9.850%; AAV SYVN1+CPP, 97.17 ± 9.094%; *F*_(2,17)_ = 0.04891, *p* = 0.9524; Figure 6C). Moreover, knockdown of Dstr SYVN1 did not affect METH CPP behaviors (PBS, 275.6 ± 39.17%; AAV-Con, 250.5 ± 32.29%; AAV-SYVN1, 252.8 ± 30.05%; *F*_(2,32)_ = 0.1621, *p* = 0.8511; Figure 6D).

## Discussion

The present study demonstrated that METH CPP formation was accompanied by a significant reduction of GABA_A_α1 expression in the Dstr. The change in GABA_A_α1 expression occurs at the post-transcription level. SYVN1, as an ERAD E3 ligase, was associated with UPS-mediated GABA_A_α1 degradation.

The GABA_A_ receptors are the major inhibitory receptors in the central nervous system and can mediate fast post synaptic inhibitory effects. The α1 subunit-typed GABA_A_ receptors are the most abundant composition subtype (Wisden et al., 1992; Fritschy et al., 1994; Liu and Wong-Riley, 2004) and appear to be correlated with drug addiction. Human genetic research demonstrated that the single nucleotide polymorphism (SNP) at rs2279020 of GABA_A_α1 subunit gene was associated with ATS-dependence (Lin et al., 2003). Liu et al. (2007) reported that continuous 7-days treatment with cocaine decreased GABA_A_ α1 expression in mouse brain induced by cocaine (Liu et al., 2007). Consistently, we found that the expression level GABA_A_α1, but not the other subunits GABA_A_α2, GABA_A_α3, GABA_A_α5 and GABA_A_β2, was decreased in the Dstr of rats after conditioned METH pairing. We previously demonstrated that the GABA_A_α1 expression level in the Dstr remained unchanged in the unpaired group, indicating the changes of GABA_A_α1 were related to METH-pairing, rather than the pure drug effects (Jiao et al., 2016). Thus, the decrease of GABA_A_α1 contributed to METH-associated rewarding memory formation. We found that the GABA_A_α1 mRNA level did no change, demonstrating that the change in GABA_A_α1 expression occurred at the post-transcription level. METH-induced down-regulation of GABA_A_α1 might cause decreased GABAergic inhibition, which triggered DA release in the Dstr, leading to a motivation for drug seeking.

ERAD is the process by which the ER directs the degradation of misfolded or inappropriate proteins (Olzmann et al., 2013; Ruggiano et al., 2014). When misfolded proteins accumulated in ER, the ER resident chaperone GRP78 increased for modifying misfolded or inappropriate proteins (Yu et al., 1999; Walter and Ron, 2011), and increased CHOP would trigger cell apoptosis if ER dysfunction was severe or prolonged. Furthermore, misfolded and inappropriate proteins would be delivered to E3 ubiquitin ligases and then ubiquitinated and degraded by the UPS. The mammalian ERAD systems were organized primarily by two E3 ubiquitin ligases: SYVN1 and Gp78 (Christianson et al., 2011). In the present study, we demonstrated that the ubiquitin-proteasome degradation via ERAD contributed to the regulation of GABA_A_α1 receptor expression during conditioned METH pairing. Several lines of evidence supported this conclusion. First, we demonstrated that conditioned METH pairing decreased GABA_A_α1 receptor expression and increased E3 ubiquitin ligase SYVN1 expression. The interactions between GABA_A_α1 receptor and SYVN1 were presented in rodent Dstr. We further showed that knockdown of SYVN1 in primary striatum neurons and rodent Dstr both increased GABA_A_α1 receptor expression level. Moreover, we found that conditioned METH pairing enhanced expression of ubiquitin protein in Dstr, and intra-Dstr injection of proteasomal inhibitor Lactacystin and MG132 reversed the changes of GABA_A_α1 expression. Thus, our data provided solid evidence supporting that UPS, as one part of ERAD, was associated with the degradation of GABA_A_α1. Li et al. (2008) and Mao et al. (2009) reported that repeated METH administration could increase ubiquitin conjugation in striatal neurons of rat (Li et al., 2008; Mao et al., 2009).

Different pathogenic factors, such as abnormal calcium regulation (Pyrko et al., 2007), viral infection (Isler et al., 2005), high-fat feeding (Deldicque et al., 2010), various mutations (Chen et al., 2013) as well as METH or cocaine abuse (Jayanthi et al., 2004, 2009; Beauvais et al., 2011; Pavlovsky et al., 2013) may disrupt ER homeostasis and cause ER stress. ER stress is associated with increased expression of the ER resident chaperone GRP78 (Yu et al., 1999) and the nuclear protein CHOP (Welihinda et al., 1999). It has been found that repeated METH administration can increase GRP78 and CHOP expression in the striatum (Jayanthi et al., 2004, 2009). However, in the present study, the expression level of GRP78 and CHOP was not altered in the Dstr. One possibility was that the dose and frequency of METH administration used in Jayanthi et al. (2004, 2009) was much higher (40 mg/kg, once a day, for seven times) than we used in the present work (1 mg/kg, once 2 days, for four times). The low dose and low frequency of METH treatment may only induce GABA_A_α1 degradation via ERAD, without affecting the level of GRP78 and CHOP. Although conditioned CPP pairing was not able to change the expression level of GRP 78 and CHOP, our study showed that *in vivo* knockdown of SYVN1 significantly increased the level of GRP 78 and CHOP in the Dstr. *In vivo* knockdown of Dstr SYVN1 increased the expression level of total GABA_A_α1 and GABA_A_α1 in the intra-ER, without affecting GABA_A_α1 level in the extra-ER. Furthermore, knockdown of Dstr SYVN1 was not able to affect METH CPP formation. It was reasonable to speculate that knockdown of Dstr SYVN1 might reduce degradation of misfolded and inappropriate GABA_A_α1, and accumulation of these misfolded protein aggregates in ER would result in ERS. Thus, increase of GABA_A_α1 induced by knockdown of Dstr SYVN1 was not able to prevent METH CPP behavior.

## Conclusion

**Figure 7:**
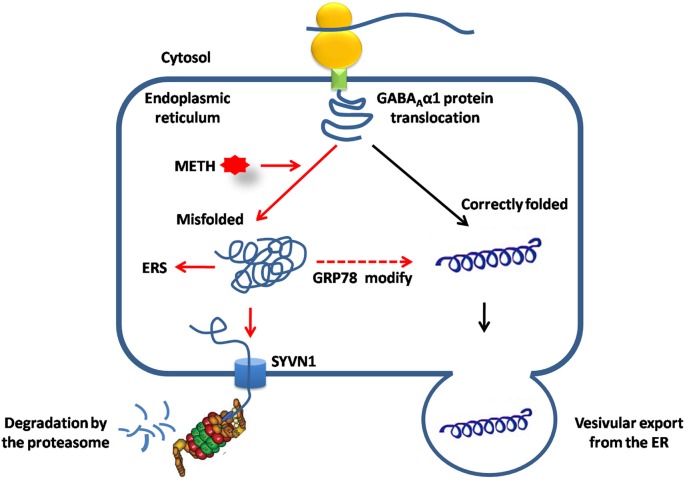
SYVN1, as the endoplasmic reticulum associated degradation (ERAD) E3 ubiquitin ligase was involved in Dstr GABA_A_α1 degradation associated with METH conditioned pairing. Normally, GABA_A_α1 proteins was correctly folded in ER, and then exported to cytosol or assembled to the cell membrane. METH treatment caused misfolded or inappropriate GABA_A_α1 accumulated in the ER, induced ERS and led to increase of GRP78 to help to modifying misfolded GABA_A_α1 proteins. Misfolded GABA_A_α1 proteins are then delivered to SYVN1 and degraded by UPS.

In summary, the present study demonstrated that the UPS via ERAD contributed to GABA_A_α1 receptor degradation during conditioned METH pairing. SYVN1, an ERAD E3 ubiquitin ligase, was the key component regulating GABA_A_α1 receptor expression (Figure 7).

## Author Contributions

MZ, Y-JW, J-GL, C-XY and D-LJ: designed experiments. D-LJ, YC, YL, J-DL and Y-YJ: carried out experiments. YL and JD: analyzed experimental results. D-LJ wrote the first draft of the manuscript. MZ, Y-JW and J-GL provided critical revision of the manuscript for important intellectual content.

## Conflict of Interest Statement

The authors declare that the research was conducted in the absence of any commercial or financial relationships that could be construed as a potential conflict of interest.

## Abbreviations

ANOVA: analysis of variance
ATS: amphetamine-type stimulants
CHOP: C/EBP homologous protein
CPP: conditioned place preference
Dstr: dorsal striatum
ER: endoplasmic reticulum
ERAD: endoplasmic reticulum associated degradation
ERS: endoplasmic reticulum stress
GABA: gamma-aminobutyric acid
Gp78: Glycoprotein 78
GRP78: Glucose-regulated protein 78
METH: methamphetamine
SYVN1: Synovial apoptosis inhibitor 1
UPS: ubiquitin-proteasome system.

## References

[B1] Addolorato G. Leggio L. Hopf F. W. Diana M. Bonci A. (2012). Novel therapeutic strategies for alcohol and drug addiction: focus on GABA, ion channels and transcranial magnetic stimulation. Neuropsychopharmacology 37, 163–177. 10.1038/npp.2011.216 22030714 PMC3238087

[B2] Batki S. L. Harris D. S. (2004). Quantitative drug levels in stimulant psychosis: relationship to symptom severity, catecholamines and hyperkinesia. Am. J. Addict. 13, 461–470. 10.1080/10550490490512834 15764424

[B3] Beauvais G. Atwell K. Jayanthi S. Ladenheim B. Cadet J. L. (2011). Involvement of dopamine receptors in binge methamphetamine-induced activation of endoplasmic reticulum and mitochondrial stress pathways. PLoS One 6:e28946. 10.1371/journal.pone.0028946 22174933 PMC3236770

[B4] Berro L. F. Andersen M. L. Tufik S. Howell L. L. (2017). GABA_A_ receptor positive allosteric modulators modify the abuse-related behavioral and neurochemical effects of methamphetamine in rhesus monkeys. Neuropharmacology 123, 299–309. 10.1016/j.neuropharm.2017.05.010 28495376 PMC5513762

[B5] Chen Y. M. Zhou Y. Go G. Marmerstein J. T. Kikkawa Y. Miner J. H. (2013). Laminin β2 gene missense mutation produces endoplasmic reticulum stress in podocytes. J. Am. Soc. Nephrol. 24, 1223–1233. 10.1681/ASN.2012121149 23723427 PMC3736718

[B6] Christianson J. C. Olzmann J. A. Shaler T. A. Sowa M. E. Bennett E. J. Richter C. M.. (2011). Defining human ERAD networks through an integrative mapping strategy. Nat. Cell Biol. 14, 93–105. 10.1038/ncb2383 22119785 PMC3250479

[B7] Curran C. Byrappa N. McBride A. (2004). Stimulant psychosis: systematic review. Br. J. Psychiatry 185, 196–204. 10.1192/bjp.185.3.196 15339823

[B8] Deldicque L. Cani P. D. Philp A. Raymackers J. M. Meakin P. J. Ashford M. L.. (2010). The unfolded protein response is activated in skeletal muscle by high-fat feeding: potential role in the downregulation of protein synthesis. Am. J. Physiol. Endocrinol. Metab. 299, E695–E705. 10.1152/ajpendo.00038.2010 20501874

[B9] Fritschy J. M. Paysan J. Enna A. Mohler H. (1994). Switch in the expression of rat GABA_A_-receptor subtypes during postnatal development: an immunohistochemical study. J. Neurosci. 14, 5302–5324. 8083738 10.1523/JNEUROSCI.14-09-05302.1994 PMC6577100

[B10] Gallagher M. J. Shen W. Song L. Macdonald R. L. (2005). Endoplasmic reticulum retention and associated degradation of a GABA_A_ receptor epilepsy mutation that inserts an aspartate in the M3 transmembrane segment of the α1 subunit. J. Biol. Chem. 280, 37995–38004. 10.1074/jbc.M508305200 16123039

[B11] Hondebrink L. Tan S. Hermans E. van Kleef R. G. Meulenbelt J. Westerink R. H. (2013). Additive inhibition of human α1β2γ2 GABA_A_ receptors by mixtures of commonly used drugs of abuse. Neurotoxicology 35, 23–29. 10.1016/j.neuro.2012.12.003 23266428

[B12] Isler J. A. Skalet A. H. Alwine J. C. (2005). Human cytomegalovirus infection activates and regulates the unfolded protein response. J. Virol. 79, 6890–6899. 10.1128/jvi.79.11.6890-6899.2005 15890928 PMC1112127

[B13] Jayanthi S. Deng X. Noailles P. A. Ladenheim B. Cadet J. L. (2004). Methamphetamine induces neuronal apoptosis via cross-talks between endoplasmic reticulum and mitochondria-dependent death cascades. FASEB J. 18, 238–251. 10.1096/fj.03-0295com 14769818

[B14] Jayanthi S. McCoy M. T. Beauvais G. Ladenheim B. Gilmore K. Wood W. III. (2009). Methamphetamine induces dopamine D1 receptor-dependent endoplasmic reticulum stress-related molecular events in the rat striatum. PLoS One 4:e6092. 10.1371/journal.pone.0006092 19564919 PMC2699544

[B15] Jiao D. Liu Y. Li X. Liu J. Zhao M. (2015). The role of the GABA system in amphetamine-type stimulant use disorders. Front. Cell. Neurosci. 9:162. 10.3389/fncel.2015.00162 25999814 PMC4419710

[B16] Jiao D. L. Liu Y. Long J. D. Du J. Ju Y. Y. Zan G. Y.. (2016). Involvement of dorsal striatal α1-containing GABA_A_ receptors in methamphetamine-associated rewarding memories. Neuroscience 320, 230–238. 10.1016/j.neuroscience.2016.02.001 26868969

[B17] Johnson B. A. Roache J. D. Ait-Daoud N. Wells L. T. Wallace C. L. Dawes M. A.. (2007). Effects of acute topiramate dosing on methamphetamine-induced subjective mood. Int. J. Neuropsychopharmacol. 10, 85–98. 10.1017/s1461145705006401 16448579

[B18] Kang J. Q. Macdonald R. L. (2004). The GABA_A_ receptor β2 subunit R43Q mutation linked to childhood absence epilepsy and febrile seizures causes retention of α1β2γ2S receptors in the endoplasmic reticulum. J. Neurosci. 24, 8672–8677. 10.1523/JNEUROSCI.2717-04.2004 15470132 PMC6729953

[B19] Kumar K. Sharma S. Kumar P. Deshmukh R. (2013). Therapeutic potential of GABA_B_ receptor ligands in drug addiction, anxiety, depression and other CNS disorders. Pharmacol. Biochem. Behav. 110, 174–184. 10.1016/j.pbb.2013.07.003 23872369

[B21] Lin S. K. Chen C. K. Ball D. Liu H. C. Loh E. W. (2003). Gender-specific contribution of the GABA_A_ subunit genes on 5q33 in methamphetamine use disorder. Pharmacogenomics J. 3, 349–355. 10.1038/sj.tpj.6500203 14569258

[B20] Li X. Wang H. Qiu P. Luo H. (2008). Proteomic profiling of proteins associated with methamphetamine-induced neurotoxicity in different regions of rat brain. Neurochem. Int. 52, 256–264. 10.1016/j.neuint.2007.06.014 17904249

[B23] Liu Q. Wong-Riley M. T. (2004). Developmental changes in the expression of GABA_A_ receptor subunits α1, α2 and α3 in the rat pre-Botzinger complex. J. Appl. Physiol. 96, 1825–1831. 10.1152/japplphysiol.01264.2003 14729731

[B22] Liu N. Y. Zhang L. Zhang L. Wang X. N. (2007). Mediating effect of dopamine D3 receptors on Jak2 and GABA_A_α1 expression in mouse brains induced by cocaine. Chin. Med. J. 120, 910–914. 17543182

[B24] Mao L. M. Wang W. Chu X. P. Zhang G. C. Liu X. Y. Yang Y. J.. (2009). Stability of surface NMDA receptors controls synaptic and behavioral adaptations to amphetamine. Nat. Neurosci. 12, 602–610. 10.1038/nn.2300 19349975 PMC2749993

[B25] Mintzer M. Z. Griffiths R. R. (2003). Triazolam-amphetamine interaction: dissociation of effects on memory versus arousal. J. Psychopharmacol. 17, 17–29. 10.1177/0269881103017001689 12680736

[B26] Olzmann J. A. Kopito R. R. Christianson J. C. (2013). The mammalian endoplasmic reticulum-associated degradation system. Cold Spring Harb. Perspect. Biol. 5:a013185. 10.1101/cshperspect.a013185 23232094 PMC3753711

[B27] Pavlovsky A. A. Boehning D. Li D. Zhang Y. Fan X. Green T. A. (2013). Psychological stress, cocaine and natural reward each induce endoplasmic reticulum stress genes in rat brain. Neuroscience 246, 160–169. 10.1016/j.neuroscience.2013.04.057 23644055 PMC3691328

[B28] Pyrko P. Kardosh A. Liu Y. T. Soriano N. Xiong W. Chow R. H.. (2007). Calcium-activated endoplasmic reticulum stress as a major component of tumor cell death induced by 2,5-dimethyl-celecoxib, a non-coxib analogue of celecoxib. Mol. Cancer Ther. 6, 1262–1275. 10.1158/1535-7163.mct-06-0629 17431104

[B29] Ruggiano A. Foresti O. Carvalho P. (2014). Quality control: ER-associated degradation: protein quality control and beyond. J. Cell Biol. 204, 869–879. 10.1083/jcb.201312042 24637321 PMC3998802

[B30] Rush C. R. Stoops W. W. Wagner F. P. Hays L. R. Glaser P. E. (2004). Alprazolam attenuates the behavioral effects of d-amphetamine in humans. J. Clin. Psychopharmacol. 24, 410–420. 10.1097/01.jcp.0000130553.55630.ad 15232333

[B31] Spence A. L. Guerin G. F. Goeders N. E. (2016). The differential effects of alprazolam and oxazepam on methamphetamine self-administration in rats. Drug Alcohol Depend. 166, 209–217. 10.1016/j.drugalcdep.2016.07.015 27485488

[B32] United Nations Office on Drugs and Crime (UNODC) (2011). “Amphetamine and ecstasy,” in The 2011 Global ATS Assessment, (New York, NY)

[B33] Walter P. Ron D. (2011). The unfolded protein response: from stress pathway to homeostatic regulation. Science 334, 1081–1086. 10.1126/science.1209038 22116877

[B34] Welihinda A. A. Tirasophon W. Kaufman R. J. (1999). The cellular response to protein misfolding in the endoplasmic reticulum. Gene Expr. 7, 293–300. 10440230 PMC6174664

[B35] Wisden W. Laurie D. J. Monyer H. Seeburg P. H. (1992). The distribution of 13 GABA_A_ receptor subunit mRNAs in the rat brain. I. Telencephalon, diencephalon, mesencephalon. J. Neurosci. 12, 1040–1062. 1312131 10.1523/JNEUROSCI.12-03-01040.1992 PMC6576059

[B36] Yu Z. Luo H. Fu W. Mattson M. P. (1999). The endoplasmic reticulum stress-responsive protein GRP78 protects neurons against excitotoxicity and apoptosis: suppression of oxidative stress and stabilization of calcium homeostasis. Exp. Neurol. 155, 302–314. 10.1006/exnr.1998.7002 10072306

